# Virological outcomes of antiretroviral therapy in Zomba central prison, Malawi; a cross-sectional study

**DOI:** 10.7448/IAS.20.1.21623

**Published:** 2017-08-02

**Authors:** Happy Mpawa, Aunex Kwekwesa, Alemayehu Amberbir, Daniela Garone, Oscar H. Divala, Gift Kawalazira, Vanessa van Schoor, Henry Ndindi, Joep J. van Oosterhout

**Affiliations:** ^a^ Dignitas International, Zomba, Malawi; ^b^ Zomba District Health Office, Malawi Ministry of Health, Zomba, Malawi; ^c^ Partners in Hope, Lilongwe, Malawi; ^d^ David Geffen School of Medicine, University of California, Los Angeles, USA; ^e^ Malawi Prison Service, Lilongwe, Malawi; ^f^ Department of Medicine, University of Malawi College of Medicine, Blantyre, Malawi

**Keywords:** HIV, prisoners, antiretroviral therapy, Malawi, adherence, viral load

## Abstract

**Introduction**: Antiretroviral therapy (ART) outcomes that include viral suppression rates are rarely reported among African prison populations. Prisoners deal with specific challenges concerning adherence to ART. We aimed to describe virological outcomes of ART in a large prison in Malawi.

**Methods**: A cross-sectional study of ART outcomes was conducted at the Zomba Central Prison HIV clinic, Malawi, following the introduction of routine viral load monitoring. All prisoners on ART for at least 6 months were eligible for a viral load test. Patients with ≥1,000 copies/ml received adherence support for 3 months, after which a second VL sample was taken. Patients with ≥5,000 copies/ml on the second sample had virological failure and started 2nd line ART. We describe demographics and patient characteristics and report prevalence of potential- and documented virological failure. In the potential virological failure rate, those who could not be sampled after 3 months adherence support are included as virological failures. Logistic regression analysis was used to determine factors associated with potential ART failure.

**Results and discussion**: Viral load testing was started at the end of 2014, when 1054 patients had ever registered on ART. Of those, 501 (47.5%) had transferred out to another clinic, 96 (9.1%) had died, 11 defaulted (1.0%) and 3 (0.3%) stopped ART. Of 443 (42.0%) remaining alive in care, an estimated 322 prisoners were on ART >6 months, of whom 262 (81.4%) were sampled. Their median age was 35 years (IQR 31–40) and 257 (98.1%) were male. Self-reported adherence was good in 258 (98.5%). The rate of potential ART failure was 8.0%, documented ART failure was 4.6% and documented HIV suppression 95.0%. No patient characteristics were independently associated with potential ART failure, possibly due to low numbers with this outcome.

**Conclusions**: Good virological suppression rates can be achieved among Malawian prisoners on ART, under challenging circumstances.

## Introduction

High rates of HIV, sexually transmitted infections and tuberculosis are found among Malawian prisoners [1,2]. The national HIV programme of the Ministry of Health has implemented antiretroviral therapy (ART) with some success in prisons [3], often under difficult circumstances of overcrowding, shortage of staff, insufficient nutrition and poor sanitation. WHO and national HIV guidelines recommend viral load (VL) monitoring for the early detection of ART failure and to improve adherence [4].

Prisoners deal with special challenges concerning adherence to treatment, including violence, confiscation of antiretroviral drugs, food insecurity, injecting drug abuse and lack of privacy, which can vary by setting and may impact on ART outcomes [5–7]. Few reports of ART outcomes in prisoners are available that include viral suppression rates. A small study of the impact of directly observed ART, conducted between 2003 and 2005, reported that 50% of American prisoners had undetectable VL 48 weeks after the intervention was introduced [8]. In Connecticut USA, 70% of 882 prisoners on ART between 2005 and 2012 had undetectable VL before release from prison [9] and achieving full viral suppression was associated with female gender and a lower psychiatric illness score. In 275 Italian prisoners on ART, optimal virological results were observed in 74% in a report from 2013 [10] and in an audit of HIV care in British prisons 68% of 74 prisoners on ART had full HIV suppression [11].

Because of a lack of studies with comprehensive ART outcomes in African prisoners we aimed to describe virological results of ART in Zomba Central Prison, Malawi and explored risk factors for virological failure.

## Methods

The Zomba Central Prison (ZCP) is a large, high security prison in southern Malawi housing around 2500 inmates. HIV prevalence among prisoners was found to be 37% in a study conducted in 2007 [12]; recent routine surveillance data indicate that prevalence is similar [unpublished data 2015]. In 2010 an HIV clinic was established at ZCP which has been supported by Dignitas International, a Canadian medical and research organization with a long-term presence in the south-east health zone of Malawi. The health care staff consists of a clinical officer, nurse and clerk. In ZCP, prisoners self-administer their ART medication. Routine VL monitoring was introduced in 2014, as per national HIV guidelines [13]. Patients should undergo VL testing at fixed time points, that is at 6 months and 2 years post ART initiation and every two years thereafter. Patients with ≥1,000 copies/ml receive intensive adherence counselling and support over a period of 3 months, after which a second VL sample is taken if adherence was reported to have been good by patient and health care worker. Patients with ≥5,000 copies/ml on the second sample have virological failure and are started on 2^nd^ line ART. Those with VL 1,000–4,999 copies/ml are subjected to a further 3-month adherence support period followed by a third VL test.

**Figure 1.**
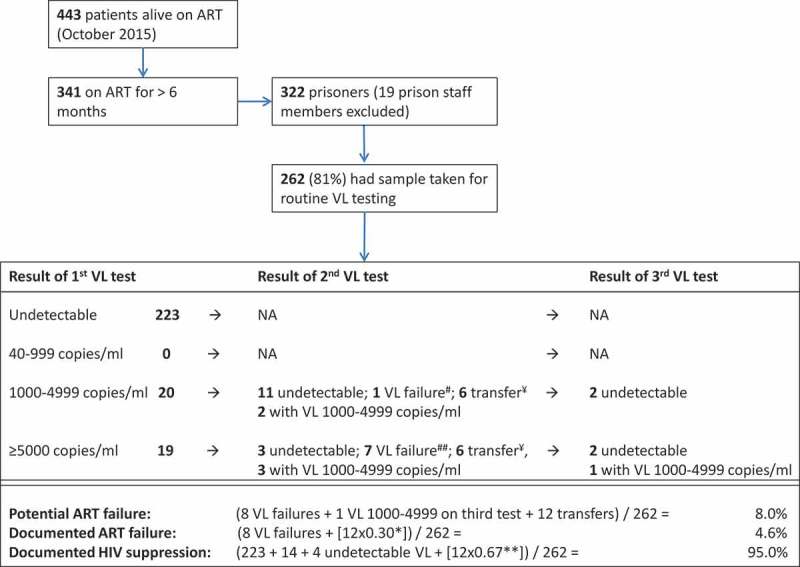
Outcomes of VL testing at Zomba Prison. Undetectable = <40 copies/mL; # = virological failure: VL result ≥ 5000 on repeat VL test after 3 months of adherence support and with good self-reported adherence. ## = virological failure according to the same definition, but includes one person who died before the second VL test could be taken. ¥ = transfer: transferred to another HIV clinic before repeat sample could be taken; NA: not applicable. * accounting for potential failures among transfers by multiplying the number of transfers with the prevalence of virological failure (30%) among those undergoing a second VL test. **accounting for potential suppression among transfers by multiplying the number of transfers with the prevalence of suppression (67%) among those undergoing a second and third VL test.

Upon first introduction of routine VL monitoring in the ZCP clinic, so-called catch-up testing was implemented: all prisoners and prison staff who were registered on ART for at least 6 months had samples taken for VL testing. We conducted a cross-sectional survey of ART outcomes and HIV-1 RNA results using routinely collected data from ART registers, HIV master cards (containing VL test results), Prison Screening Cards and quarterly HIV clinic reports. Prison staff members on ART who are also registered in the clinic were excluded from our analyses. We used the following definitions: *potential ART failure*: (those found with virological failure + those who transferred to another clinic before a second VL could be done + those who had a VL result between 1,000 and 4,999 on the third sample)/those tested; *documented ART failure*: (those found with virological failure + [those who transferred out times the prevalence of VL >5,000 copies/ml among those undergoing a second VL test])/those tested; *documented HIV suppression*: (those found with an undetectable VL+ [those who transferred out times the prevalence of undetectable VL among those undergoing a second and third VL test]) /those tested (Figure 1). A defaulter was defined as in the national HIV guidelines [7]: a patient who has not returned to the clinic two months after he/she is expected to have run out of medication and whose outcome is unknown. If patients are released from prison they transfer to a regular HIV clinic. If they move to another prison, they transfer to an HIV clinic at that prison if present or otherwise to a nearby clinic that provides HIV services for the prison. Samples were taken between November 2014 and June 2015 and processed at the molecular laboratory at Zomba Central Hospital with the Abbott m2000, Real-time HIV-1 system®, detection level 40 copies/ml (Abbott Laboratories, Abbott Park, Illinois, U.S.A.). Undetectable VL is therefore defined as <40 copies/mL. We entered data in an anonymized database and conducted descriptive analyses of demographic and clinical characteristics of the study participants. Multivariable logistic regression analysis was used to assess independent risk factors of the outcome potential ART failure, using the likelihood ratio test for significance testing. Analyses were done with Stata 13 software (Statacorp, College Station Texas, USA). Because we analysed anonymized, routinely collected HIV programme and prison health service data, individualized informed consent was not obtained. The study was approved by the College of Medicine Ethics and Research Committee, Blantyre, Malawi (P.08/16/2014).

## Results and discussion

We first describe facility-level ART outcomes based on the third HIV clinic quarterly report of 2014 [14]. The number of patients who had ever been registered on ART was 1054, of whom 501 (47.5%) were transferred to another clinic, 96 (9.1%) died, 11 (1.0%) defaulted, 3 (0.3%) stopped ART and 443 (42.0%) were alive in care at the ZCP clinic.

When routine VL monitoring was first introduced we estimated that 341 patients were on ART >6 months and thus eligible for VL testing. Nineteen prison staff members who were registered at the clinic were excluded from the analysis. We were able to take samples from 262/322 (81.4%) patients. Of those sampled, the median age was 35 years (IQR 31–40) and 257 (98.1%) were male. At the start of ART, 145 (55.6%) were in WHO stage 3 or 4 and 45 (19.0%) had prevalent tuberculosis. Of the 92 patients who had a recorded CD4 count at ART initiation, 34 (37.0%) had a value <200 cells/µL. The current ART regimen for 92.8% of patients was tenofovir/lamivudine/efavirenz, 2.7% were on zidovudine/lamivudine/nevirapine and 4.6% on another regimen. The median duration of ART at the time of sampling for VL tests was 32 months (IQR 15–48). Self-reported adherence during the last clinic visit was good in 258 (98.5%). Among prisoners, the median self-reported duration of incarceration was 4 years (IQR 2–5). The outcomes of VL testing are presented in Figure 1. In a multivariable logistic analysis of factors associated with potential ART failure, there was no significant association with age, WHO clinical stage and tuberculosis treatment status at ART initiation, duration of incarceration, current ART regimen, and ART duration. CD4 count at ART initiation was left out of the model due to missing data, and gender, current ART regimen and self-reported adherence due to very low numbers of females, on non-standard first-line regimens and with poor adherence respectively (Table 1). We report good ART outcomes in the HIV clinic of a large prison in Malawi. A low cumulative rate of defaulting (1%) was observed, which is remarkably lower than the national rate (21%). A moderate cumulative rate of death was similar to the national percentage (9%) [14]. Among those who were in care, virological suppression was excellent, documented ART failure being 4.6% and potential ART failure 8.0%. Due to the introduction of VL monitoring and adherence support for those with high VL results, the prevalence with undetectable VL was increased by 6.9%, bringing it above the 90% UNAIDS target level for virological suppression [15].

**Table 1:** Multivariable analysis of factors associated with potential ART failure^§^ in the HIV clinic of ZCP

	Potential ART failure			
Characteristics	n (%) potential ART failure	Crude OR (95% CI)	Adjusted OR (95% CI)	P-value*
*Gender (N=262)*				-
Male	21 (8.2)	-	-	
Female	0 (0.0)	-	-	
*Age in years (N=262)*				0.75
19-34	10 (7.8)	1	1	
≥35	11 (8.3)	1.07 (0.44, 2.62)	1.17 (0.46, 2.92)	
*WHO stage at ART initiation (N=261)*				0.39
WHO stage 1	5 (4.6)	1	1	
WHO stage 2	1 (16.7)	4.20 (0.41, 43.04)	4.63 (0.44, 48.80)	
WHO stage 3	14 (10.2)	2.39 (0.83, 6.86)	2.33 (0.71, 7.66)	
WHO stage 4	1 (12.5)	3.00 (0.31, 29.31)	3.32 (0.30, 36.33)	
*Prevalent tuberculosis^¥^ (N=262)*				0.47
No	14 (7.3)	1	1	
Yes	3 (6.7)	0.91 (0.25, 3.30)	0.61 (0.15, 2.49)	
Missing	4 (16.0)	2.42 (0.73, 8.04)	1.68 (0.46, 6.21)	
*CD4 Count at ART initiation (N=92)*				
CD4 <200	6 (17.7)	-		
CD4 ≥200	0 (0.0)	-		
*Current ARV regimen (N=262)*				
tenofovir/lamivudine/efavirenz	20 (8.2)	1		
zidovudine/lamivudine/nevirapine	0 (0.0)	-		
tenofovir/lamivudine/nevirapine	1 (8.3)	1.01 (0.12, 8.26)		
*ART duration in months (N=261)*				0.85
6-24	7 (7.1)	1	1	
≥24	14 (8.6)	1.22 (0.48, 3.14)	1.10 (0.40, 3.04)	
*Duration incarceration years (N=262)*				0.43
<4	12 (9.5)	1	1	
≥4	9 (6.6)	0.67 (0.27, 1.66)	0.68 (0.27, 1.75)	
*Adherence (N=281)*				
Good	21 (8.1)	-	-	
Poor	0 (0.0)	-	-	

ART, antiretroviral therapy; ZCP, Zomba Central Prison; OR, Odds ratio; WHO, World Health Organization

OR’s are adjusted for age, WHO disease stage, Prevalent tuberculosis, ART duration and duration of incarceration.

The CD4 count (missing data), current ART regimen (very small numbers with ART failure category), gender (very few female prisoners) and adherence (very low number with poor adherence) variables were omitted from the multivariable model.

**^§^**Potential ART failure (N=21) is defined as those found with VL failure (n=8) + those with VL>1,000 on the first sample and who transferred before second sample could be taken (n=12) plus those with VL 1,000-4,999 copies/ml on 2 repeated tests (n=1)

*Based on the likelihood ratio test

*^¥^*Prevalent tuberculosis is tuberculosis diagnosis at ART initiation

We found no other studies that documented virological outcomes of prisoners from Malawi and only one from sub-Saharan Africa. In a South African prison, losses to follow up among 148 prisoners were high (22%) both during incarceration and after release but in the 70 patients who remained in care, 92% had an undetectable VL [16]. Our virological results and those of the South African study compare favourably with reports from prisons in affluent settings where rates of undetectable VL were between 50 and 74% [8–11] as well as with recent routinely collected VL data from the whole population on ART in Malawi, showing that 11% of VL results were >1,000 copies/ml [17].

Our virological ART outcomes are particularly encouraging given that prisoners face many challenges that affect adherence and increase the risk of ART failure. A recent systematic review and meta-analysis of adherence among prisoners on ART that included 11 studies (10 from the USA and Europe and a conference abstract of a small study from Kenya), found that only 55% of prisoners had good adherence as measured by self-reported indicators and pharmacy refill data. Drug abuse was the only individual factor that was significantly associated with poor adherence [5]. In a Namibian study, prisoners mentioned multiple factors that hindered adherence such as lack of privacy, presence of stigma, absence of a clock, insufficient food, brutality in the prison setting, low motivation to stay healthy, and poor general knowledge of HIV [7]. Unfortunately these qualitative findings were not corroborated with actual adherence data and virological outcomes. Although some of those factors also prevail in Malawian prisons, we found that rates of self-reported good adherence and virological suppression were high. The presence of an HIV clinic on the prison premises and the secluded patient population facilitate tracking potential defaulters which may have contributed to the good ART results.

Limitations of our study are important to consider. First, 18% of the patients on ART were not sampled for VL testing and the sampled population therefore may not have been fully representative of the prison population on ART. Prisoners transfer in and out of the HIV clinic frequently due to movements between prisons and release from custody and transfers are the most likely reason for missed samples when VL testing was first introduced. In addition, some patients may have avoided VL testing due unwillingness to give a blood sample. Prisoners who stopped ART and defaulters are assumed not to have a suppressed VL. However only three patients defaulted or stopped ART during the study period, therefore this did not contribute to non-sampling relevantly, thus had minimal impact on suppression rates and is an important reason why virological success is unlikely to be overestimated in our study. Second, we did not provide an overview of the total HIV care cascade as data on HIV prevalence were incomplete at the time of the study. Third, it is probable that small numbers of patients with the outcome potential ART failure precluded finding significant associations with patient characteristics and clinical variables. Future studies may provide adequate power to find such associations if they exist. Lastly, we have no follow up information of patients after transfers out of the prison clinic, when linkage to care can be problematic [16].

## Conclusion

Our study shows that good ART results can be achieved in sub-Saharan prisoners under challenging circumstances.
